# Successful interventional closure of patent ductus arteriosus in three pediatric cases with congenital heart disease and severe pulmonary hypertension: a case series and literature review

**DOI:** 10.3389/fcvm.2025.1628666

**Published:** 2025-07-09

**Authors:** Xiong-Yu Liao, Jun-Jie Li, Shao-Ying Zeng, Zhi-Wei Zhang, Yu-Mei Xie

**Affiliations:** ^1^ Department of Cardiology, Children’s Medical Center, Sun Yat-sen Memorial Hospital, Sun Yat-sen University, Guangzhou, China; ^2^ Department of Pediatric Cardiology, Guangdong Cardiovascular Institute, Guangdong Provincial People’s Hospital (Guangdong Academy of Medical Sciences), Southern Medical University, Guangzhou, China

**Keywords:** PDA, severe PH, Eisenmenger syndrome, targeted therapy, interventional closure

## Abstract

**Purpose:**

To report three cases of successful closure of patent ductus arteriosus (PDA) with severe pulmonary hypertension (PH) and explore interventional closure strategies for congenital heart disease (CHD) complicated by severe PH. This study aims to determine whether such patients can undergo and benefit from interventional closure, providing clinical insights for physicians.

**Methods:**

A retrospective analysis was conducted on three pediatric cases of PDA with severe PH successfully treated via interventional closure at the Department of Pediatric Cardiology, Guangdong Provincial People's Hospital. Literature related to “patent ductus arteriosus,” “severe pulmonary hypertension,” “Eisenmenger syndrome,” “targeted therapy,” and “interventional closure” (in both Chinese and English) was searched in PubMed and the China National Knowledge Infrastructure (CNKI) database up to November 2024. Case characteristics and therapeutic strategies were analyzed in conjunction with the literature.

**Results:**

All three pediatric patients had CHD combined with severe PH and underwent successful PDA occlusion after administration of at least 3 months of endothelin receptor antagonist or PDE5 inhibitor therapy (targeted therapy). Postoperative targeted therapy was continued, with follow-up until November 30, 2024. No significant elevation in pulmonary artery pressure (PAH) was observed, and exercise tolerance markedly improved in all cases.

**Conclusion:**

Children with CHD and severe PH may qualify for interventional closure after a period of targeted therapy and could benefit from this procedure. Administering targeted medications before and after closure not only provides opportunities for intervention but also reduces the risk of persistent postoperative PAH.

## 1 Introduction

Patent ductus arteriosus (PDA), a common congenital heart defect (CHD) characterized by left-to-right shunting, has an incidence of approximately 0.5% and accounts for 5%–10% of all CHD cases (1). PDA can lead to increased pulmonary vascular resistance and elevated pulmonary artery pressure (PAP). A large PDA may result in severe pulmonary hypertension (PH), progressing to Eisenmenger's syndrome (ES), right heart failure, or even death (2). In developing countries, including China, inadequate early screening and treatment protocols for CHD in certain regions have contributed to a substantial population of children with CHD-associated PH. Many left-to-right shunting CHD cases evolve into ES, thereby missing the optimal window for surgical intervention (3, 4). Consequently, determining therapeutic strategies for CHD-PH patients—such as evaluating surgical indications, postoperative management, and assessing potential benefits from corrective procedures—has become a critical focus for clinicians. While prior studies established PDA closure safety in moderate PH, evidence for interventional management in children with severe PH following targeted therapy remains limited. This study retrospectively analyzes three pediatric cases of PDA with severe PH successfully treated via interventional closure at the Department of Pediatric Cardiology, Guangdong Provincial People's Hospital, and reviews relevant literature to provide insights for the diagnosis and management of CHD-PH in children. We present the following article in accordance with the MDAR reporting checklist.

## 2 Methods and materials

### 2.1 Collection of clinical data from three pediatric cases with PDA and severe PH successfully treated via closure

Clinical data were retrospectively collected and analyzed from three pediatric patients diagnosed with PDA and severe PH who underwent successful PDA closure and received targeted medical therapy at the Department of Pediatric Cardiology, Guangdong Provincial People's Hospital, between January 2024 and August 2024. All three patients had compound cardiac malformations combined with severe PH. PDA is confirmed by 2D echocardiography and color Doppler. Severe PH defined as measured mPAP ≥ 50 mmHg + PVRi > 8 WU-m^2^ by right heart catheterization. Dynamic PAH was defined as a ≥20% reduction in PVR on pulmonary vascular reactivity testing and fixed PAH was defined as irreversible elevation of pulmonary vascular resistance (2022 ESC/ERS Guidelines). Two or three targeted drugs were administered for at least 3 months after PDA closure, with subsequent adjustments based on the patient's echocardiography (e.g., RV systolic function, PAP trends) or cardiac catheterization findings as well as on cardiac function and activity tolerance. Response criteria: Primary: ≥20% reduction in PVR or PAP on catheterization. Secondary: Improved functional class (NYHA/Ross) and 6-minute walk distance [6MWD]. Data included medical history, laboratory findings, imaging studies (echocardiography, chest x-ray), cardiac catheterization results, and postoperative outcomes. Outcomes assessed via NYHA class, SpO_2_, NT-proBNP, and 6MWD (age ≥5 years). This study was approved by the Ethics Committee of Guangdong Provincial People's Hospital (KY2025-126-01) and exempted from informed consent.

### 2.2 Literature review

A comprehensive literature search was conducted using the keywords “patent ductus arteriosus” “severe pulmonary hypertension” “Eisenmenger's syndrome” “targeted therapy,” and “interventional closure” (in both Chinese and English) in PubMed and the China National Knowledge Infrastructure (CNKI) database. Articles published up to November 2024 were screened to identify and analyze reported cases of PDA with severe PH successfully managed via interventional closure.

## 3 Results

### 3.1 Clinical data of three pediatric cases with PDA and severe PH successfully treated via closure

#### 3.1.1 Case details

##### 3.1.1.1 Case 1

**Figure 1:**
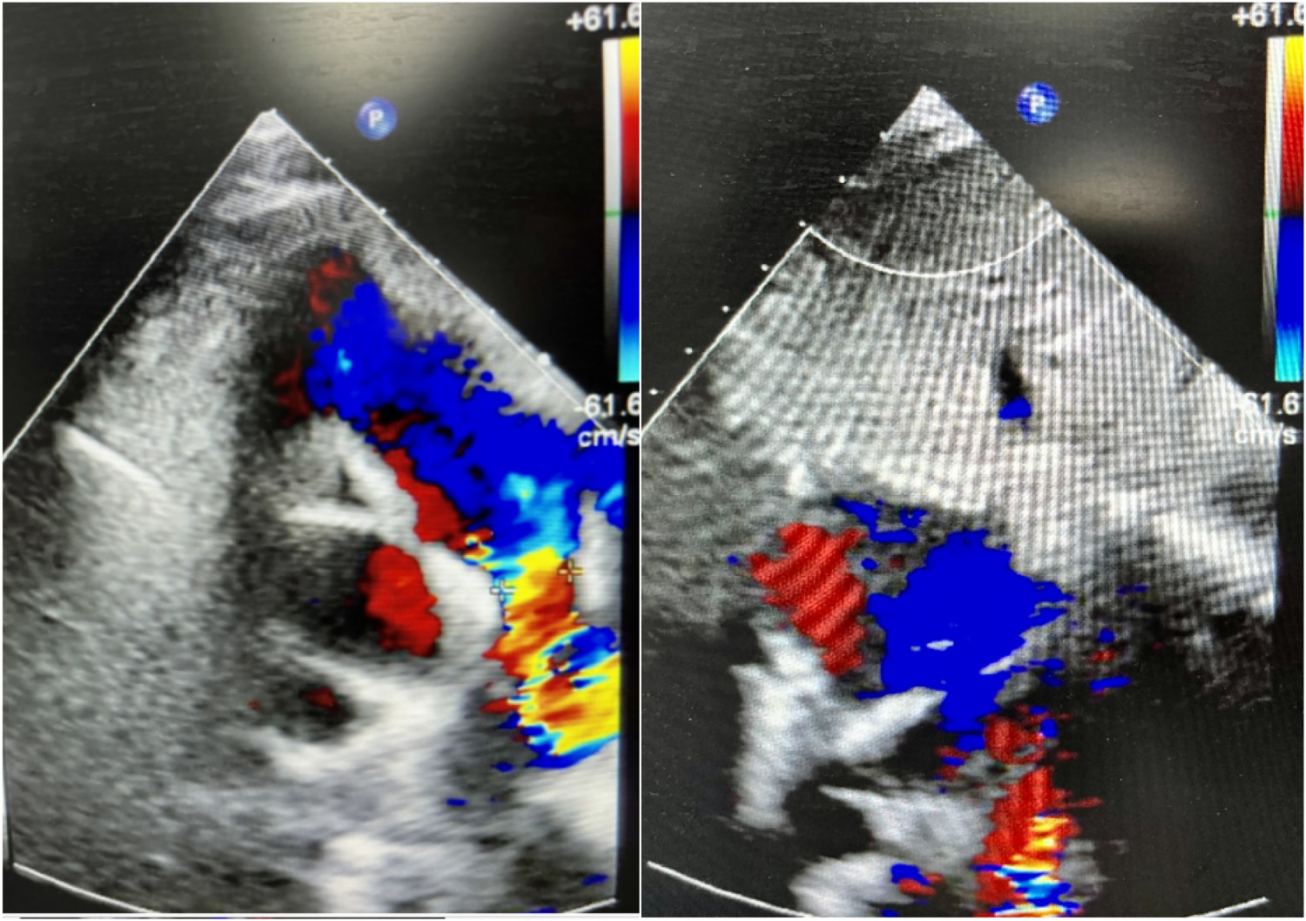
Cardiac ultrasound of case 1 at guangdong provincial people's hospital (January 11, 2024).

**Figure 2:**
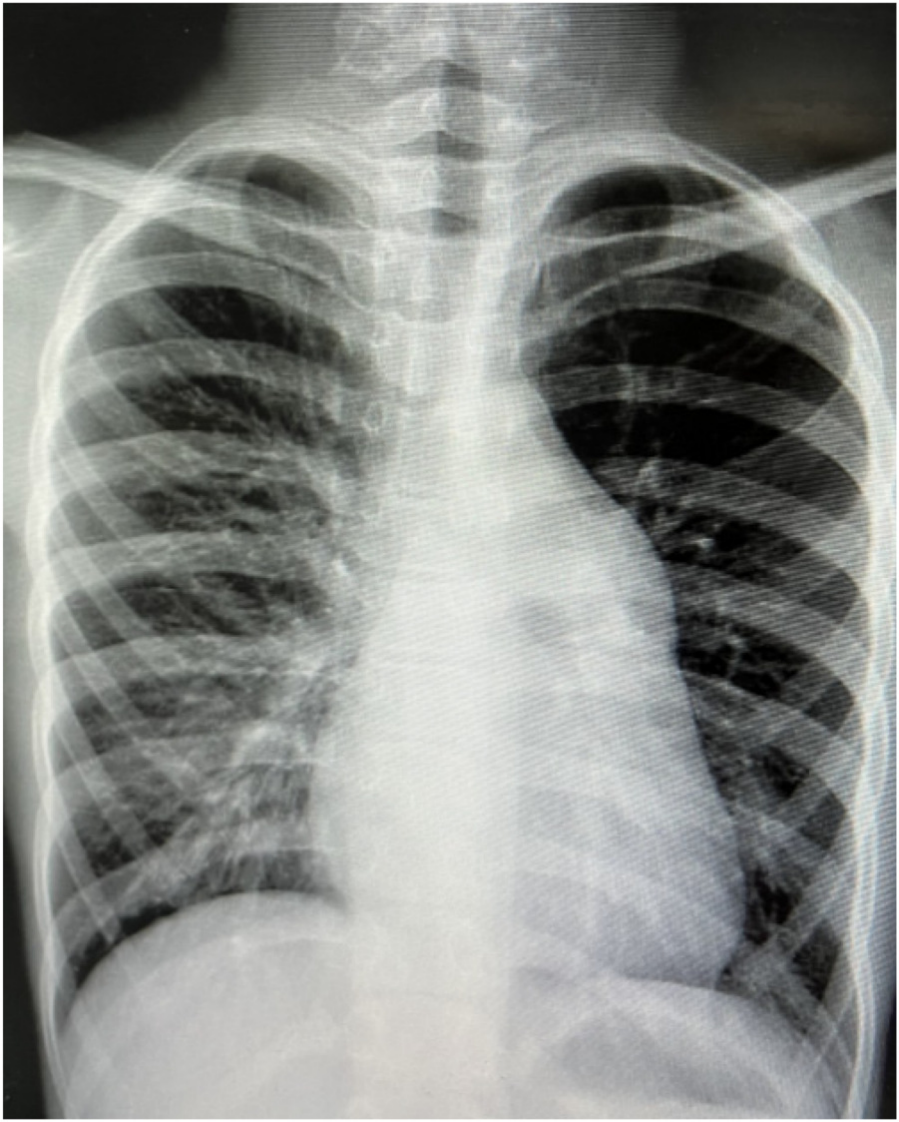
Chest x-ray of case 1 (January 11, 2024).

A 9-year-old female presented with a history of a cardiac murmur detected at age 1. Initial echocardiography (March 2016) revealed PDA, atrial septal defect (ASD), and PH. She reported symptoms including occasional dyspnea, reduced exercise tolerance compared to peers, post-exertional wheezing, squatting preference, cyanosis of the lips and lower extremities, and clubbing of fingers. Two prior attempts at PDA closure (May 2016 at another institution, and April 2018) were aborted due to persistently elevated pulmonary artery pressure (PAP) despite targeted therapy with endothelin receptor antagonists (ERA) and phosphodiesterase-5 (PDE5) inhibitors, initiated after the first failed attempt. Therapy evolved over time, including periods of triple therapy (ambrisentan, sildenafil, treprostinil) and dual therapy (ambrisentan + sildenafil), adjusted due to side effects and financial constraints. Upon admission to our center (January 2024) (Table 1, Figure 1, 2), physical examination revealed differential cyanosis (upper limb SpO2 90%–92%, lower limb SpO2 80%–82%), cyanotic lips, clubbing, an accentuated P2 heart sound, but no pathological murmurs. Echocardiography confirmed a large tubular PDA (10.3 mm, predominantly right-to-left shunt), secundum ASD (6.4 mm, bidirectional shunt), severe PH (estimated sPAP 97 mmHg), mild tricuspid regurgitation, and evience of right ventricular hypertrophy and dysfunction (TAPSE 11 mm, FAC 33%) (Table 2). Cardiac catheterization (March 2024) under general anesthesia confirmed precapillary PH (mPAP 57 mmHg, PCWP 9 mmHg, PVRi 11.24 WU-m^2^, descending aortic SpO2 77%, Qp/Qs 1.32). Following successful trial occlusion demonstrating a significant reduction in mPAP (to 45 mmHg) and normalization of descending aortic SpO2 (100%), a 20/22 mm Lifetech PDA occluder was deployed and real-time hemodynamic monitoring metrics (mPAP, PVR, SaO_2_). Postoperatively, triple therapy (sildenafil, ambrisentan, treprostinil) was continued. Follow-up at 3 months (June 2024) and 5 months (August 2024) post-closure showed marked clinical improvement: resolution of cyanosis (lower limb SpO_2_ 91%–94%), improved exercise tolerance (6MWD) increased from 239 m pre-op to 333 m and then 390 m) (Table 3), and stable or slightly improved echocardiographic parameters (sPAP 100 mmHg at 3 m, 93 mmHg at 5 m; improved RV function TAPSE 19.7 mm/15.6 mm). The ASD remained patent.

**Table 1:** Longitudinal echocardiographic parameters of the case 1.

Date	Heart chamber changes	ASD size/shunt	sPAP (mmHg)	PDA status/shunt	LVEF (%)	TAPSE (mm)	FAC (%)	E′/A
Dec 2016	Right heart enlargement	PFO, Bidirectional	65	Bidirectional shunt	64.2	–	–	–
Sep 2017	Right heart enlargement	8 mm, L → R dominant	79	7 mm, Bidirectional	73.7	–	–	–
Dec 2017	Right heart enlargement	7 mm, L → R dominant	79	7 mm, Bidirectional	69.1	–	–	–
Apr 2018	Right heart enlargement	7 mm, L → R dominant	67	7 mm, Bidirectional	65.0	–	–	–
Mar 2023	Right heart enlargement	7 mm, Bidirectional	79	9 mm, Bidirectional	88.0	–	–	–
Jan 2024	RA 38 mm, RV hypertrophy (24 mm)	6.4 mm, Bidirectional	97	10.3 mm, R → L dominant	86.0	11.0	33	3
Mar 2024a	Right heart enlargement	10.5 mm, Bidirectional	87	9.7 mm, R → L dominant	79.0	–	–	–
Mar 2024b	RA enlargement, RV hypertrophy	11 mm, L → R dominant	98	Closed (no residual)	77.0	–	–	–
Jun 2024	RA enlargement, RV hypertrophy	10–16 mm, L → R dominant	100	Closed (no residual)	77.0	19.7	35	3
Aug 2024	RA enlargement, hypertrophy	13–16 mm, L → R dominant	93	Closed (no residual)	80.0	15.6	28	3

L-R denotes left-to-right shunt; R-L denotes right-to-left shunt; RA, right atrium; RV, right ventricle.

**Table 2:** Cardiac catheterization: pulmonary artery pressure, blood oxygen saturation of the descending aorta, and hemodynamic parameters.

Cases	Time	PAP (mmHg)	Qp (L/min)	Qs (L/min)	Qp/Qs	PVR (Wood U)	SpO2 (DAo)
Case 1	September 2017	98/56 (72)	4.83	5.15	0.94	12.22	96.4%
	March 2018	88/44 (64)	9.47	6.03	1.57	5.28	99.3%
	Pre-occlusion PDA trial on March 12, 2024	84/45 (57)	5.61	4.25	1.32	11.24	97%
	Post-occlusion PDA trial on March 12, 2024	67/36 (45)	3.94	3.77	1.04	14.97	100%
Case 2	Pre-occlusion PDA trial on July 25, 2024	82/37 (52)	8.54	3.32	2.57	9.94	97%
	Post-occlusion PDA trial on July 25, 2024	81/28 (45)	6.09	2.47	2.47	8.94	100%
	Pre-occlusion ASD trial on October 25, 2024	86/34 (51)	5.64	3.58	1.58	9	100%
	Post-occlusion ASD trial on October 25, 2024	68/21 (38)	5.0	3.48	1.45	7.52	100%
Case 3	Pre-occlusion PDA trial on August 15, 2024	97/52 (68)	10.4	4.7	2.2	8.3	100%
	Post-occlusion PDA trial on August 15, 2024	82/51 (61)	6.9	5.2	1.3	11.2	100%

**Table 3:** Patient 1—Six-minute walk test.

Date	Walking distance (m)	Respiration score	Lower limb score	Heart failure severity assessment
January 12, 2024 (Pre-occlusion)	239	0	9–10	Moderate
June 12, 2024 (3 months post-operation)	333	0	9–10	Moderate
August 20, 2024 (over 5 months post-operation)	390	0	6–8	Moderate

##### 3.1.1.2 Case 2

A 14-year-old female with Trisomy 21 (Down syndrome) and residing in a welfare institution was diagnosed with PDA, secundum ASD, persistent left superior vena cava (LSVC), and severe PH via echocardiography (January 2024). She was initiated on targeted therapy (sildenafil + bosentan) and admitted to our center in February 2024. Her symptoms included reduced exercise tolerance but no dyspnea, cyanosis, edema, syncope, or hypoxic episodes. Physical examination revealed cyanotic lips, an accentuated P2 heart sound, no pathological murmurs, and no clubbing. Limb SpO2 was symmetric and normal (98%–99% upper limbs, 96% lower limbs). Echocardiography confirmed a tubular PDA, secundum ASD, severe PH, mild tricuspid regurgitation, and LSVC. Cardiac catheterization (July 2024) confirmed severe precapillary PH (mPAP 52 mmHg, PCWP 6 mmHg, PVRi 9.94 WU-m^2^, descending aortic SpO2 97%, Qp/Qs 2.57) (Table 3). Trial PDA occlusion resulted in a favorable hemodynamic response (mPAP decrease to 45 mmHg, aortic pressure increase, SpO2 100%), and a 10/12 mm Lifetech PDA occluder was successfully deployed and real-time hemodynamic monitoring metrics (mPAP, PVR, SaO_2_). Targeted therapy was transitioned to tadalafil + macitentan. Three months later (October 2024), a second catheterization confirmed persistent PH (mPAP 51 mmHg, PVRi 9 WU-m^2^) with Qp/Qs 1.58. Following a positive response to ASD trial occlusion (mPAP decrease to 38 mmHg), the ASD was closed with a 30 mm Lifetech occluder. The patient remained stable on dual targeted therapy post-operatively.

##### 3.1.1.3 Case 3

A 10-year-7-month-old male was admitted in August 2024 with a 10-month history of chest tightness and dyspnea following strenuous activity. Physical examination revealed cyanotic lips, an accentuated P2 heart sound, no pathological murmurs, and no clubbing. Limb SpO2 was normal and symmetric (99%–100%). Echocardiography identified a large perimembranous ventricular septal defect (VSD) with bidirectional shunting, a funnel-shaped PDA, moderate-to-severe tricuspid regurgitation, mild pulmonary valve regurgitation, severe PH, and persistent LSVC. Cardiac catheterization (August 2024) confirmed severe precapillary PH (mPAP 68 mmHg, PCWP 12 mmHg, PVRi 8.3 WU-m^2^, Qp/Qs 2.2) (Table 3). Trial PDA occlusion resulted in a reduction of mPAP (to 61 mmHg) and maintenance of systemic saturation (SpO2 100%), allowing successful deployment of a 12/14 mm Lifetech PDA occluder. Post-operatively, triple targeted therapy (sildenafil + bosentan + treprostinil) was initiated. At the 3-month follow-up (November 2024), the patient exhibited markedly improved exercise tolerance and resolution of cyanosis. Echocardiography estimated sPAP at 87 mmHg. The VSD remained patent. Triple therapy was continued.

#### 3.1.2 Treatment and outcomes summary

All three pediatric patients with CHD (PDA plus ASD or VSD) and severe PH underwent successful transcatheter PDA closure following a period of pre-operative targeted medical therapy (at least 3 months). Targeted therapy was continued post-operatively. Follow-up evaluations conducted until November 30, 2024 (ranging from 1 to 6 months post-closure), demonstrated significant clinical improvement in all cases. Key positive outcomes included markedly improved exercise tolerance and subjective well-being. Critically, echocardiography performed during follow-up did not reveal any significant elevation in estimated PAP compared to pre-closure or immediate post-closure levels. Case 1 notably showed resolution of cyanosis in the lips and lower extremities, increased peripheral oxygen saturation, and a substantial improvement in 6-minute walk distance. These findings suggest that PDA closure, when performed following targeted therapy and guided by favorable intra-procedural hemodynamic response, is feasible and associated with beneficial clinical outcomes in this challenging patient population with severe PH and multiple shunts. Short-term follow-up of all three patients showed stable results, further long-term follow-up is ongoing.

### 3.2 Literature review findings

A systematic literature search identified six case reports documenting successful transcatheter closure in pediatric patients with Eisenmenger syndrome (ES), predominantly involving isolated PDA. The majority of these reported cases also utilized targeted therapy both before and after the closure procedure, aligning with the management strategy employed in our case series.

## 4 Discussion

Historically, many scholars have posited that dynamic pulmonary arterial hypertension (PAH) in children with CHD-PAH is amenable to surgical correction, whereas fixed PAH is considered a contraindication (1, 5). However, there remains no universally accepted international standard to definitively distinguish between dynamic and fixed PAH. Current PAH guidelines recommend using pulmonary vascular resistance (PVR) measured during cardiac catheterization as a critical determinant for surgical candidacy in CHD-PAH (2). Widely accepted thresholds include PVR <4–6 Wood units (WU) and pulmonary-to-systemic blood flow ratio (Qp/Qs) > 1.5, whereas the suitability of surgery for patients with Qp/Qs 1.0–1.5 remains controversial (3). In our study, Case 1 had previously undergone multiple failed PDA closure attempts at other institutions, mainly due to high PVR and low Qp/Qs (<1.5). Notably, perioperative factors such as hyperventilation or hypoventilation under general anesthesia may transiently alter PAP and PVR, complicating hemodynamic assessments.

Yan et al. reported successful PDA closure in 20 children with severe PH (mean PASP 104 mmHg, mean PVR 9.1 WU), using a ≥25% reduction in PASP after trial occlusion as the criterion for definitive closure (6). A single-center study in China documented three successful PDA closures in patients with Eisenmenger syndrome (ES), despite Qp/Qs <1.5 and PVR >15 WU (7). Thanopoulos et al. achieved successful PDA closure in seven patients with Qp/Qs ≥2.0, demonstrating a ≥30% post-occlusion PASP reduction (8). In our cohort, the mean pre-closure PASP was 88 mmHg. During trial occlusion at Guangdong Provincial People's Hospital, Case 1 exhibited a near 30% reduction in PASP, and all three cases maintained stable aortic SpO_2_, indicating no acute hemodynamic compromise post-occlusion. Interestingly, postoperative Qp/Qs was unchanged but mPAP decreased in case 2, which may be associated with abnormal remodeling of the pulmonary vasculature and alveolar dysplasia in patients with trisomy 21 (citing trisomy 21 PH literature).These findings suggest that CHD-PAH patients with Qp/Qs <1.5 and high PVR may not completely lose surgical candidacy. Further studies with larger cohorts are warranted to refine optimal interventional criteria.

Extensive evidence highlights that targeted PAH therapy improves exercise capacity, reduces PVR, and promotes pulmonary vascular remodeling through endothelin-1 inhibition, activation of the NO-cGMP pathway, and antiproliferative effects, resulting in safe occlusion (1, 9, 10). A case series of four PDA-ES patients with PVR >15 WU demonstrated favorable outcomes after one year of preoperative targeted therapy followed by PDA closure and continued postoperative pharmacotherapy (11). In our study, preoperative targeted therapy in Cases 1 and 2 facilitated successful PDA closure, with Case 2 subsequently undergoing ASD closure three months later. All three patients maintained triple PAH therapy postoperatively, achieving improved exercise tolerance and stable echocardiography-estimated PAP during follow-up. Sustained Postoperative Benefit Suggests permanent vascular remodeling. Cases 1 and 3 resumed schooling with enhanced quality of life (QoL) which was objectively measured via NYHA class, 6MWD and school reintegration. These outcomes challenge traditional paradigms, suggesting that resistance PAH in CHD may retain residual pulmonary vascular reactivity, allowing surgical intervention after targeted therapy (7, 10, 12). Postoperative pharmacotherapy may further mitigate the risk of persistent PAH (12, 13). This study suggests that the primary goal of managing severe PAH in CHD should focus on functional improvement and quality of life, rather than merely reducing PAP. Functional status and QoL are primary endpoints in severe CHD-PH management. It is noteworthy that unlike other studies that have only one malformation of the PDA, all cases in this group had two left-to-right shunts including the PDA. Our strategy prioritized PDA closure to mitigate both volume overload (from increased pulmonary blood flow) and pressure overload (from aortic-to-pulmonary shunting) (2, 14). Residual ASD in Cases 1–2 and VSD in Case 3 were intentionally preserved to reduce acute right ventricular strain and prevent PH crises. Post-closure targeted therapy was maintained to stabilize hemodynamics. Although PVR may not decrease as well in the short term, it may not be the only factor that we need to focus on. PDA closure may be more meaningful if we consider it from the perspective of improving the patient's activity tolerance and quality of life mentions. Whether the treatment strategy of this study can be applied to patients with severe PH caused by VSD and ASD remains to be further studied.

We have to acknowledge several limitations in this present study. First, the small sample size limits generalizability. Second, follow-up duration was short, and PAP was estimated via echocardiography rather than invasive catheterization. Short-term follow-up results needs to be validated in large cohorts monitored for ≥5 years. Future studies should validate interventional criteria for CHD-PAH and assess long-term outcomes.

## Data Availability

The raw data supporting the conclusions of this article will be made available by the authors, without undue reservation.
